# Effects of soil water and heat relationship under various snow cover during freezing-thawing periods in Songnen Plain, China

**DOI:** 10.1038/s41598-018-19467-y

**Published:** 2018-01-22

**Authors:** Qiang Fu, Renjie Hou, Tianxiao Li, Ruiqi Jiang, Peiru Yan, Ziao Ma, Zhaoqiang Zhou

**Affiliations:** School of Water Conservancy & Civil Engineering, Northeast Agricultural University, Harbin, Heilongjiang 150030 China

## Abstract

In this study, the spatial variations of soil water and heat under bare land (BL), natural snow (NS), compacted snow (CS) and thick snow (TS) treatments were analyzed. The relationship curve between soil temperature and water content conforms to the exponential filtering model, by means of the functional form of the model, it was defined as soil water and heat relation function model. On this basis, soil water and heat function models of 10, 20, 40, 60, 100, and 140 cm were established. Finally, a spatial variation law of the relationship effect was described based on analysising of the differences between the predicted and measured results. During freezing period, the effects of external factors on soil were hindered by snow cover. As the snow increased, the accuracy of the function model gradually improved. During melting period, infiltration by snowmelt affected the relationship between the soil temperature and moisture. With the increasing of snow, the accuracy of the function models gradually decreased. The relationship effects of soil water and heat increased with increasing depth within the frozen zone. In contrast, below the frozen layer, the relationship of soil water and heat was weaker, and the function models were less accurate.

## Introduction

Seasonally frozen soil is widespread in northeastern, northwestern and northern China. It is present along the edges of permafrost regions, and its freezing and thawing characteristics correspond to a state of discontinuous freezing^1^. The soil freezing and thawing cycle affects water and heat transfer, water phase changes and salt migration, simultaneously, and affects soil water evaporation and infiltration runoff processes, thereby, it changes the atmospheric water vapor cycle^2–4^. Soil freezing and thawing cycle is an important natural process of material exchange and energy transfer. It plays central roles in agricultural production, water resource development and utilization, engineering construction and environmental protection^5–8^. Investigating the migration patterns and interactional mechanisms between soil water and heat that occur during seasonal freezing and thawing periods not only contributes to theories of water migration and laws of heat diffusion in unsaturated zones in cold areas, but also reveals seasonal variations in soil moisture and heat, and promotes the advancement of related disciplines^9–11^.

Researchers in China and elsewhere have conducted numerous experimental studies examining the relationship between soil hydrothermal transfer and coupling interactions in seasonally frozen soil. For example, Bo^12^ studied the factors that influence frozen soil infiltration and found that infiltration decreased as the soil moisture content increased and that cumulative infiltration rates varied as a power function of the soil moisture content. Jame^13^ reported that soil moisture and heat in frozen soil moved downward with the frozen front, and the water content and temperature of the frozen soil are reduced. Wang^14^ observed that the temperature of seasonally freezing and thawing soils tended to increase as the ambient temperature warmed and that the ranges of soil temperature variations gradually decreased with increasing soil depth. Endrizzi^15^ used the GEOtop fine network model to predict soil moisture and heat under snow cover. This model described the processes of energy exchange between soil moisture and the atmosphere in three dimensions while taking the effects of radiation and turbulent flux into account. Nagare^16^ studied the effects of soil freezing on soil moisture content and temperature redistribution: based on simulations of freezing conditions in the laboratory, it was concluded that the freezing front exerts a certain driving effect on the soil moisture content. Liu^17^ used KD2 Pro to test the thermal conductivity of gley silty loam and found that the latent heat transfer of water vapor was greater at high temperatures; as the soil temperature increased, the soil heat transfer appeared to be greater. Li^18^ reported that the positions of water samples in different soil intervals during freezing and thawing periods displayed a non-normal distribution and observed positive partial or negative partial correlations. Due to the phase transitions of soil moisture, soil moisture was more active during melting than during freezing periods. Fu^19^ studied the interactions between soil water and heat beneath snow cover of various depths during freezing and thawing periods and concluded that the relationship between soil water and heat gradually strengthened with increased snow cover during freezing periods. However, during melting periods, this relationship decreased with increased snow cover. Li^20^ tested unsaturated soil water diffusivity via a one-dimensional soil column experiment and concluded that the temperature gradient moisture diffusion rate is proportional to the temperature.

Ground surface cover has important effects on soil freezing and melting processes and on dynamic changes of soil water and heat: it suppresses the loss of soil heat, reduces the evaporation of water, increases soil moisture, and controls the concentrations of nutrients and salt. Flerchinger^21^ studied changes in soil temperature and water content in areas with various types of vegetation and other surface cover. It was found that surface cover hindered energy exchange between the soil and the environment, reduced the loss of soil heat during freezing period, and promoted energy, water and moisture conservation. Sauer^22^ concluded that ground cover could inhibit the evaporation of shallow soil moisture, weaken the migration and distribution of water in soil, and alleviate soil salt accumulation during freezing periods. Sharratt^23^ studied the effects of various cover treatments on the freezing depth, freezing rate and freezing period of seasonally frozen soils. It was found that the freezing characteristics varied in a predictable way depending on the amount of coverage. Yang^24^ reported that the depth and the time of high water content and low value appearance of the moisture content in soil treated with surface film mulching differed from those of bare land during soil freezing and thawing. Li^25^ concluded that changes in vegetation cover had significant effects on water phase transitions during soil freezing and thawing, and influenced by plant transpiration and evaporation from the ground surface, the moisture content of the soil profile showed the migration of soil moisture upward and downward during thawing. Fu^26^ concluded that snow cover inhibited the effects of environmental factors on soil temperature and moisture, and the effect was more pronounced with increasing soil depth. Chang^27^ elucidated a significant Boltzmann function between the soil temperature and water levels in the active permafrost layer, changes in the surface cover and climate leaded to changes in the hydraulic relationship between groundwater and river water, and then affect the hydrological change process of the whole basin. Previous studies have focused on thermal transport, environmental response and thermal dynamic simulations of freezing - thawing soil. In this study, the effects of soil hydrothermal relationship and its variation at spatial scale are explored from the freezing and melting periods.

To explore soil water and heat transfer behavior and its relationship effect, we examined the dynamic patterns and spatial distributions of soil water and heat during the freezing and melting of a black soil in the Songnen Plain in northeastern China, analyzed the spatial variability of soil water and heat relationship effects under various conditions, and the influences of different snow cover treatments on the fitting and prediction effect of soil water and heat function model were investigated. This paper described the synergistic effects between water and heat in frozen soil, established equations describing the soil-cover-atmosphere system and described dynamic simulations of the soil water and heat. The ultimate goal is to understand the relationship effects of water and heat in frozen soil, to predict the soil moisture content during spring sowing and to realize the effective regulation of soil water and heat in the Songnen Plain, China.

## Materials and Methods

### Overview of the study area

**Figure 1:**
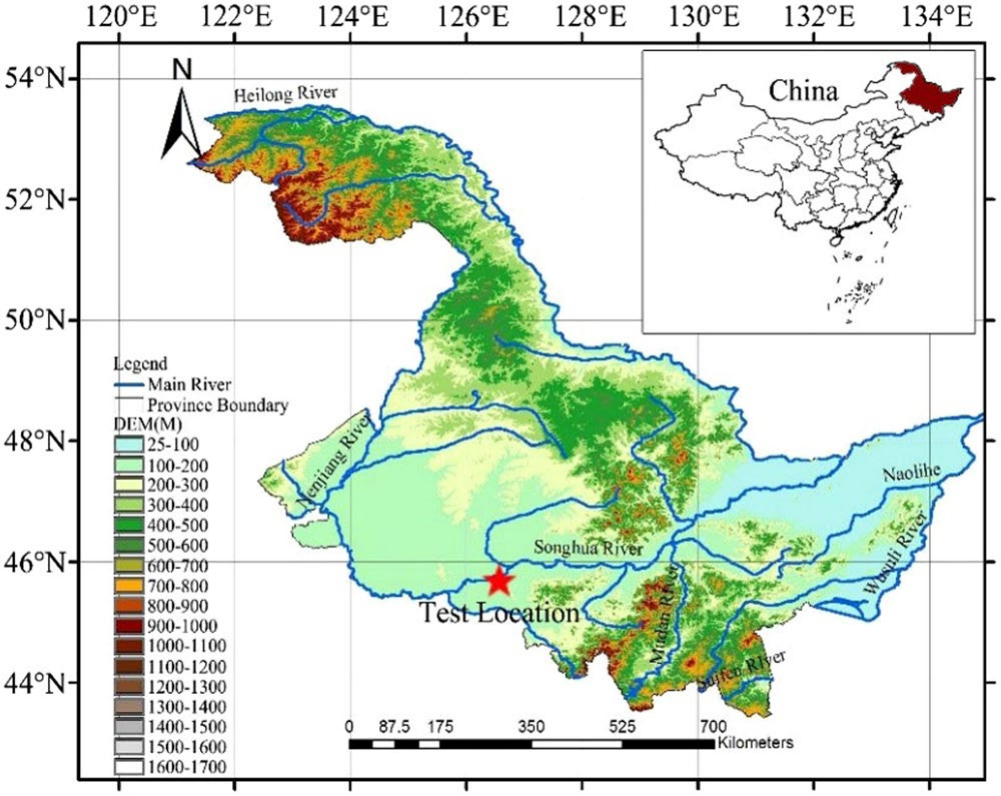
Location of the study area. The map and the inset map in this image were drawn by the authors using Arcgis software, and the elevations were derived from a DEM. The software version was Arcgis software v. 10.2, and its URL is http://www.esri.com/.

The study area is located in the Northeast Agricultural University Water Comprehensive Test Field in northeastern China’s Songnen Plain at E126°45′32″, N45°44′41″. The soil type in this study area is typical of the region, which is shown in Fig. 1. The study area is flat, and the soil nutrients are stable. Sampling indicates that the plowed interval extends to a depth of approximately 30 cm and that the soil is black soil. The soil between depths of 30 cm and 60 cm is a clay loess, and the soil layer below 60 cm consists of clay. The soil’s physical properties are listed in Table 1. Average annual precipitation in the area is 560 mm, average annual evaporation is 1,326 mm, the total volume of water resources is 9.6 billion cubic meters, and the annual average temperature is 2–6 °C. The area has a temperate continental monsoon climate. The local winters and summers are short, and spring and autumn temperatures change quickly. The local precipitation falls mainly during 6–9 months of the year, during which approximately 60% of the annual precipitation occurs. Local winter snowfall occurs mainly from November to January of the following year. The minimum temperatures occur in January, and the annual average minimum temperature is −19.6 °C. The maximum temperatures occur in July, and the highest average temperature is 22.4 °C. The average annual duration of snow cover is 110 days. Our sampling indicates that the soil mechanical properties in the area are uniform and stable.

**Table 1:** Soil physical properties.

Soil depth, cm	Bare land		Natural snow		Compacted snow		Thick snow	
	Field water holding rate, %	Soil dry bulk density, g/cm^3^	Field water holding rate, %	Soil dry bulk density, g/cm^3^	Field water holding rate, %	Soil dry bulk density, g/cm^3^	Field water holding rate, %	Soil dry bulk density, g/cm^3^
10	30.21	1.25	32.64	1.35	32.51	1.34	29.21	1.32
20	31.28	1.22	32.18	1.33	33.24	1.32	31.24	1.35
40	32.61	1.32	31.16	1.36	30.71	1.36	28.54	1.37
60	29.46	1.33	29.87	1.39	31.19	1.40	31.24	1.39
100	32.94	1.35	33.61	1.39	32.76	1.37	33.12	1.38
140	31.75	1.38	30.19	1.42	30.91	1.42	32.71	1.44

### Test setting

For the experiment, the test area was divided into four areas consisting of bare land (BL), natural snow (NS), compacted snow (CS), and thick snow (TS). Each test block measured 10 × 10 m, and test setups were constructed consisting of 2 time-domain reflectometry (TDR) probes (designated probes No. 1 and No. 2) and soil temperature sensors (JL-04, Qingshengdianzi, Handan, Hebei, China). The water contents (i.e., liquid moisture contents) and soil temperatures at depths of 10, 20, 40, 60, 100, and 140 cm were measured during freezing and melting periods. To prevent the horizontal migration of water between test plots and soil hydrothermal mutual driving effects, a 1-m-tall sheet of waterproof high-density polyethylene film was placed at the boundaries between the test plots. In each test area, a frozen soil device (LQX-DT, Jinzhoulicheng, Jinzhou, Liaoning, China) was placed and used to measure changes in the freezing depth during freezing and thawing periods. According to our observations, the maximum freezing depths in the BL, NS, CS and TS plots were 127, 118, 95, and 86 cm, respectively.

After each snowfall, four plots were individually arranged. In the BL plot, snow was removed after each snowfall to keep the ground bare. In the NS plot, the natural snowfall was left as is, and the snow cover eventually reached a stable thickness of 15 cm, at which time its density was 0.137 g/cm^3^. In the TS plot, after each snowfall, artificial snow was placed and was compacted under the weight of a board measuring 2.5 m × 1.5 m × 0.02 m and 9.8 kg/cm^2^ to ensure that the snow was compacted evenly during the tamping process. During the period of stable snow cover, its thickness was 15 cm, and its density was 0.212 g/cm^3^. In the TS plot, clean snow from outside the test plots was placed to simulate natural snow such that its density was approximately that of the natural snow. The resulting artificial snow cover was 30 cm thick, and its density was 0.140 g/cm^3^. During the tests, the snow thickness was monitored daily at 9 a.m. using a steel plate. The density and water content of the snow were measured using a Snow Fork Snow Characteristic Analyzer (Helsinki University, Finland). A probe thermometer was used to measure the snow temperature. The average value was determined after repeating the measurements 3 times. The physical characteristics of the snow are listed in Table 2.

**Table 2:** Physical parameters of the snow cover.

Treatment	Snow temperature, °C				Snow water content, %				Snow density, g/cm^3^			
	upper	middle	bottom	mean	upper	middle	bottom	mean	upper	middle	bottom	mean
Natural snow	−11.9	−10.2	−9.8	−10.6	28.3	21.4	26.9	25.5	0.127	0.157	0.127	0.137
Compacted snow	−10.5	−9.3	−8.1	−9.3	38.2	31.2	33.1	34.2	0.197	0.231	0.208	0.212
Thick snow	−10.1	−9.8	−7.6	−9.17	31.5	28.7	27.3	29.2	0.129	0.137	0.151	0.139

### Research methods

To study the soil water and heat relationship effects of the soil in this seasonally frozen area, the soil hydrothermal migration and variations law of the freezing and melting periods were examined, respectively. The SPSS software was used to analyze and manage the data. It can be found that the liquid water content of soil showed a decreasing process with the decreasing of soil temperature through the trend analysis, what’s more, the curve which corresponds to the numerical point of moisture and temperature in the form of an S-shaped curve, on this basis, the point that the soil temperature and the water content correspond one to one was fitted into the relation function. During the freezing and melting processes, the fitting function models of soil temperature and water content under the different treatment conditions were similar and can be approximated as follows^28,29^:

1 $${\theta }_{{\rm{v}}}={{\rm{A}}}_{c}/\{1+\exp [B(T-{\rm{\Delta }}{T}_{0})]\}+{\rm{\Delta }}{\theta }_{0},$$

where *A*_c_, *B*, Δ*T*_0_ and Δθ_0_ are variables whose values are affected mainly by the cover conditions and the soil temperature and moisture content.

After analyzing the curve fitting effects, we noted that the function model form conformed to an exponential filtering model. In the manuscript, the fitting effect of the mathematical function model was used to determine the relationship effect between soil water and heat during freezing and thawing periods, and it was defined as a function model of soil water and heat relationship. The MATLAB (2010b) data regression analysis toolbox was used to develop the function model relating soil moisture content at point No. 1 and temperature under the four treatment conditions at depths of 10, 20, 40, 60, 100, and 140 cm, respectively. R^2^ represents the fitting intensity effect of the model, at the same time, the relative errors (RE) between the fitting values and measured values were calculated using the SPSS software. The RE is close to 0, which indicates that the intensity of the fitting effect is good.

In addition, we conducted a statistical analysis of the moisture content and temperature data from point No. 2. According to the soil moisture content data of the No.2 test points, the soil temperature was calculated by the mathematical function model constructed above. Then, we compared the predicted soil temperature with the measured temperature, and calculated the error effect between the two, the accuracy of the model’s prediction was verified. The effective coefficient (NSE) was used to measure how well the model can predict the observation, it reflected the prediction accuracy of the model. When the NSE approaches 1, the predicted result of the model is more accurate. The NSE value is calculated as follows^30^:

2 $$NSE=[\sum _{i=1}^{n}({x}_{i}-\overline{x}{)}^{2}-\sum _{i=1}^{n}{({x}_{i}^{^{\prime} }-{x}_{i})}^{2}]/\sum _{i=1}^{n}({x}_{i}\,-\,\overline{x}{)}^{2},$$

where *x*_*i*_ is the predicted value, ${x}_{i}^{^{\prime} }$ is the measured value, and $\overline{x}$ is the average of the measured values.

## Results and Discussion

### Regularities of soil moisture variations during freezing and thawing periods

In the seasonally frozen soil region, soil freezing and melting processes were accompanied by water and heat migration. During the freezing period, the soil temperature decreased, and some liquid water froze. As the water content decreased, the soil matrix potential decreased, and under the action of the soil matrix gradient, water was driven to the freezing front^31^. During the melting period, ice in the soil melted. Under the effects of the water gravity gradient, soil moisture drove a spatial re-distribution phenomenon^32^.

**Figure 2:**
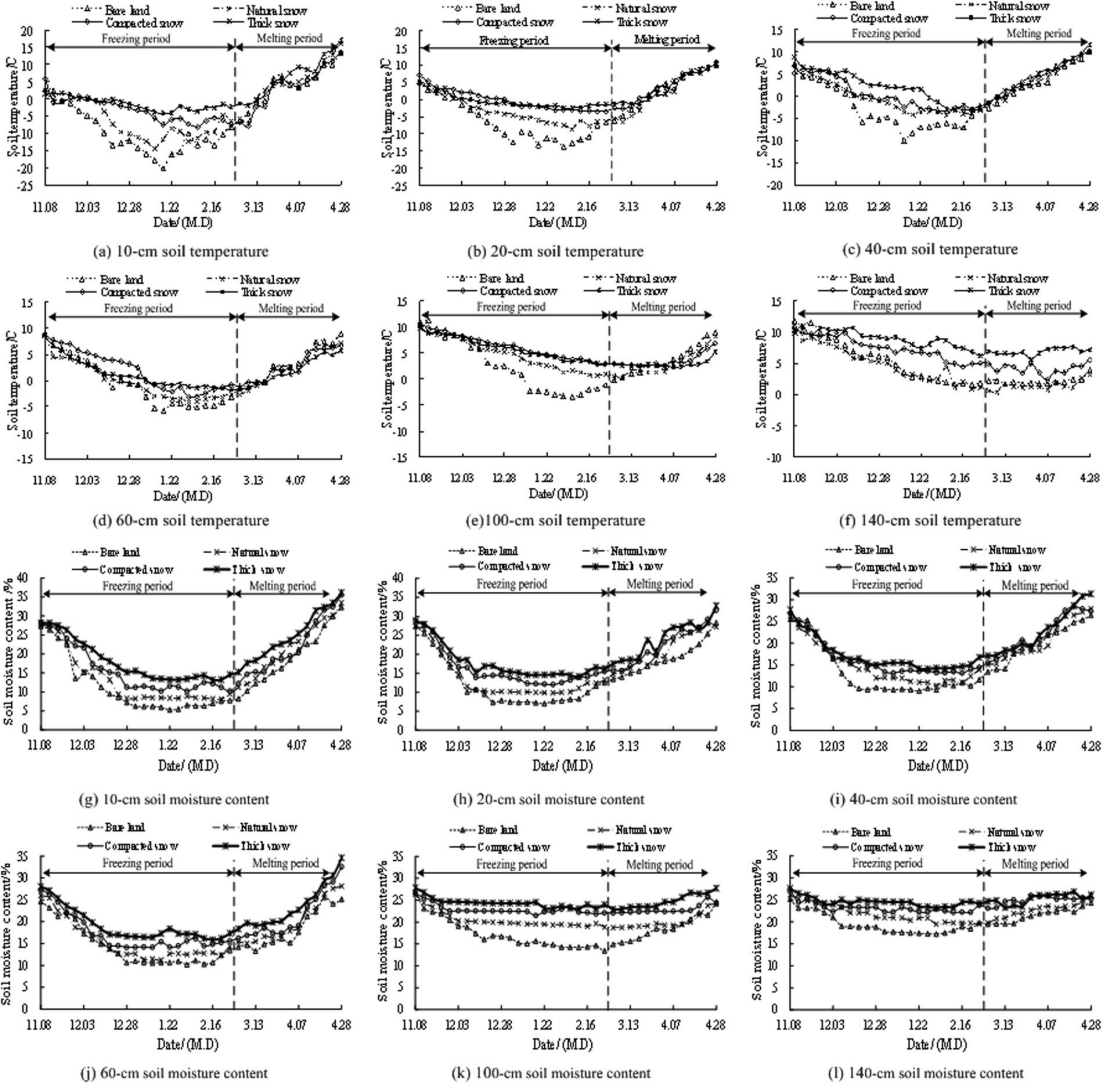
Soil temperature and water content variations in different soil layers.

These changes in soil moisture content and temperature during the test period are shown in Fig. 2. Figure 2(a) and (g) showed that along the surface at a depth of 10 cm, the variation of soil water and heat showed the trend of decreasing in freezing period and increasing in melting period under the four snow cover treatments, and the change amplitudes of the two were pronounced. During freezing period, the temperature change in the BL plot was the most significant, and the soil temperature range (ΔT) was 18.9 °C. Under the NS treatment, the temperature range was 12.7 °C, whereas under the CS and TS, which had a thicker and denser snow cover, the temperature ranges of the soil were 11.5 and 9.7 °C, respectively. Similarly, the soil moisture content ranges were 22.04%, 19.26%, 17.93% and 14.69% under the BL, NS, CS and TS treatments, respectively. Additionally, we found that as the snow cover thickness and density increased, the degrees of change decreased. During the melting period, the infiltration of snowmelt under snow cover affected soil temperature and water content variations. Therefore, fluctuations of the soil temperature and water content under the NS, CS and TS were greater than those under the BL. Analysising showed that under the BL condition, the soil temperature range was 16.7 °C, but it steadily increased. Although the soil temperature ranges under the three snow cover conditions were less significant, the variation curves fluctuated considerably. In addition, the water content increasing amplitude under the BL, NS, CS and TS were 22.02%, 24.39%, 25.35% and 27.34%, respectively, and all increased.

Based on the changes in soil temperature and water content in the 20-cm soil layer during the freezing period (Fig. 2(b) and (h)), the soil temperature and moisture content also decreased significantly. However, compared to the variations in the 10-cm soil layer, those in the deeper soil interval were smaller, the fluctuations of the two trends were reduced, and the trends in the soil water and heat variation were similar. The soil temperature difference under the BL was 18.5 °C. For comparison, the temperature differences under the NS, CS, and TS were 11.9 °C, 10.5 °C and 6.7 °C, respectively. The temperature difference declined as the snow cover increased and exhibited more stable changes than those in the 10-cm soil layer. The soil moisture content reflected the same effect. During the melting period, this parameter was also affected by snowmelt infiltration, and both soil water and heat showed complex changes. However, these complex trends were less than those in the surface (10-cm) soil layer, which exhibited a soil temperature increasing amplitude of 16.5 °C under the BL. In addition, the soil temperature increasing amplitude under the three types of snow cover (NS, CS, and TS) were 15.3 °C, 12.8 °C and 11.7 °C, respectively. The soil moisture content increased by 18.52% under the BL condition, and the increasing value was reduced by 3.5% compared with that in the 10-cm soil layer. The increasing amplitude in the water content under the NS, CS and TS were 19.37%, 20.56% and 22.68%, respectively, and the changes were more stable and showed varying degrees of decrease relative to those in the 10-cm soil layer.

**Figure 3:**
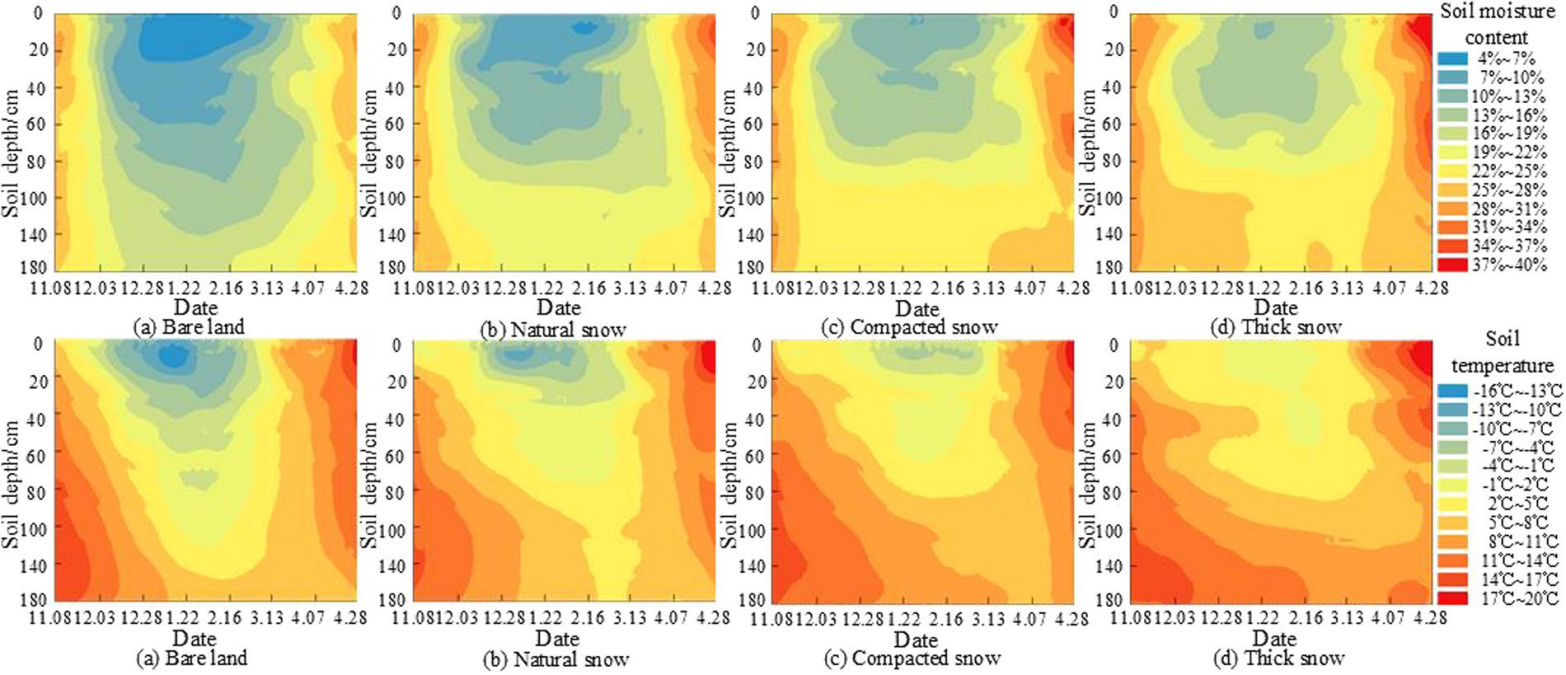
Soil water and heat variation map in the spatial during the freezing and thawing period The map was drawn by the authors using Arcgis software, and the software version was Arcgis software v. 10.2, and its URL is http://www.esri.com/.

Similarly, the changes in soil temperature and water content in the 40, 60 and 100 cm soil layers were analyzed. During the freezing period, the trends of soil temperature and water content were consistent with those of the previous soil analysis trend, but the overall variation was less than that in the 10 cm and 20 cm soil layers. As the snow cover thickened, the variation differences decreased. During the melting period, the soil temperature and water content changed significantly under the snow. Moreover, in the 140-cm soil layer, the impacts of freezing and melting were minor due to the depth, and thus changes in the soil temperature and moisture content were minor. We found soil temperature variations of 1.3–10.2 °C under the BL during the freezing period. Similarly, the variations were 1.9–9.7 °C, 2.1–9.2 °C and 3.5–8.7 °C under the NS, CS and TS treatments, respectively. The water content ranges under the four treatments were 17.69–25.18%, 20.96–25.21%, 22.25–26.56% and 24.51–27.64%, respectively. Differences in the soil temperature and water contents under the different treatments were not obvious. During the melting period, the influence of snowmelt water on the soil was limited, and changes in the soil water content under three different snow treatments were minor and constant. In addition, the spatial variations of soil water and heat in the freezing and thawing periods are shown in Fig. 3.

According to the variation of soil moisture and temperature under the conditions of different snow cover treatments, the differences under different treatments were explored in combination with the soil thermal conductivity, water conductivity and snow insulation and snow melt flow characteristics. During the freezing period, the coverage of snow hindered the energy exchange between soil and environment. With the increase of snow cover thickness and density, this heat insulation ability was enhanced, therefore, under the conditions of BL, NS, CS and TS treatments, the temperature range of soil decreased in turn. At the same time, the decrease of soil temperature gradient reduced the degree of phase change and the amount of migration of soil moisture, and the change range of soil moisture gradually weakened. During the melting period, the snow melted and produced a certain amount of snowmelt water, with the increase of snow cover, the promote of snowmelt runoff and the increase of cumulative infiltration rate of soil moisture greatly affected the variation rule of soil moisture, and further changed the thermal conductivity of soil. Therefore, in this period, with the increase of snow cover, the change of soil moisture was greater, and the fluctuation of temperature was stronger.

### Spatial variability of soil water and heat coupling during the freezing period

In the study, based on the soil freezing process curve under the natural snow treatment, it was defined that the initial melting time of the surface soil was the demarcation point of the soil freezing and thawing periods. After studying the soil freezing process, we found that the initial melting time of the surface soil under this treatment was February 10th; thus, this date was used as the dividing point between the two periods. In an earlier stage of statistical analysis, a one-time sampling analysis revealed no significant differences in soil dry density, particle composition, saturated water content and field water holding capacity under the different treatments. During the study, the effects of soil texture on soil moisture and heat transfer were negligible.

**Figure 4:**
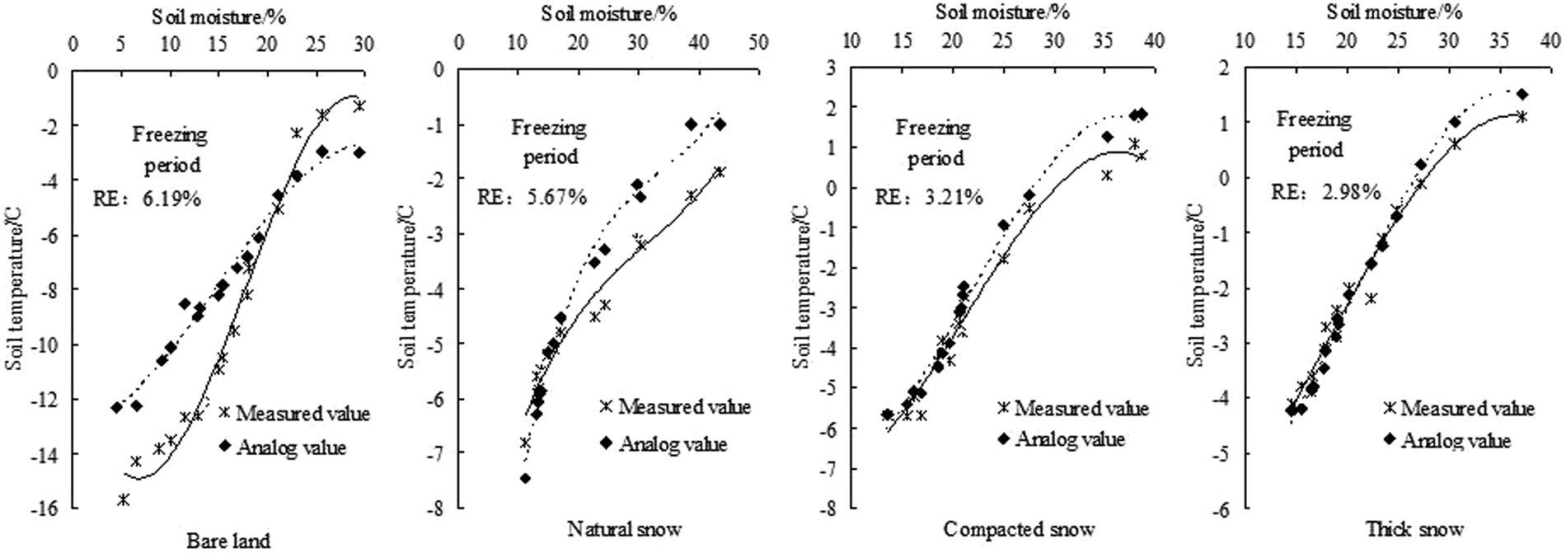
Comparison of values predicted by the soil water-heat coupling model and observed for the 10-cm surface layer during the freezing period.

We first developed a function model of soil water and heat using soil moisture content and temperature data from point No. 1 in the 10-cm soil layer. We then compared the values predicted by the model with the measured values and plotted the results in Fig. 4. The figures showed that the relationship effects were strong during the freezing period. The coefficient of determination exceeded 0.8, and through the significance test of P < 0.05, the correlation between variables was high. However, we found some differences in the relationship between soil water and heat under the different snow cover conditions. In addition, R^2^ represented the fitting intensity effect of the function model, the relative errors in Table 3 represented the error between fitting values and measured values, and the effective coefficient (NSE) was used to evaluate the agreement between the predicted and measured values. Under the BL condition, the coefficient of determination (R^2^) between the soil temperature and water content was 0.813, and those under the NS, CS and TS were 0.834, 0.875 and 0.917, respectively. The snow cover dampened the effects of external factors on the soil moisture content and temperature changes. Therefore, the fitting intensity effect of soil water and heat function model was enhanced in turn.

**Table 3:** Relationship models of soil water and heat in the 10-cm frozen soil layer during the freezing period.

	Treatment	Model of soil water and heat	Coefficient of determination, R^2^	P value	RE/%	NSE
Freezing phase	Bare land	*θ*_*V*_ = 12.31 + 17.64/(1 + exp(−(*T*_*s*_ + 3.81)/5.24))	0.813	0.0132	6.19	0.828
	Natural snow	*θ*_*V*_ = 13.74 + 21.30/(1 + exp(−(*T*_*s*_ + 4.27)/1.27))	0.834	0.0239	5.67	0.867
	Compacted snow	*θ*_*V*_ = 15.67 + 12.74/(1 + exp(−(*T*_*s*_ − 2.18)/1.52))	0.875	0.0415	3.21	0.894
	Thick snow	*θ*_*V*_ = 18.54 + 41.75/(1 + exp(−(*T*_*s*_ − 0.78)/0.56))	0.917	0.0064	2.98	0.909

According to the prediction results of the soil water and heat function models under the four treatment conditions in Fig. 4, the error between the measured and predicted values was lower between −4 °C and −8 °C, under the BL. During freezing, as the soil temperature gradually decreased, the prediction error increased, and the effective coefficient (NSE) of the model reached 0.828. Under the NS condition, the model’s prediction performance improved. During the low-temperature period, the measured and predicted values matched well, and the NSE of the model was 0.867. As the snow cover thickness and density increased, the relationship effects gradually strengthened, and the model’s fitting accuracy and predictions were more accurate under the CS and TS treatments.

After analyzing the effect of soil water and heat relationship at the soil surface (10 cm) soil layer, we developed soil water and heat function models for the 20-, 40-, 60-, 100- and 140-cm soil layers under the various treatment conditions, and the function models’ prediction effects were tested. The results are shown in Table 4. First, the relationship effects of the soil water and heat function model of the 20-cm soil layer under the four treatments were compared, and they showed trends that were generally similar to that of the 10-cm layer. Under the BL treatment, the coefficient of determination (R^2^) was 0.838, and the coefficients of determination for the three types of snow cover were significantly greater than that of the BL treatment, in the following order: TS > CS > NS. Thus, snow cover protected soil from external forces, and the relationship between soil moisture and temperature was enhanced.

**Table 4:** Effects of the soil water and heat relationship models under different treatments during the freezing period. “**” denotes passing of the P < 0.01 significance test, and “*” denotes passing of the P < 0.05 significance test.

Soil depth	Bare land			Natural snow			Compacted snow			Thick snow		
	R^2^	RE/%	NSE	R^2^	RE/%	NSE	R^2^	RE/%	NSE	R^2^	RE/%	NSE
20 cm	0.838*	2.76	0.841	0.871*	2.41	0.861	0.914*	2.09	0.871	0.959*	1.71	0.901
40 cm	0.851*	1.09	0.873	0.889*	1.31	0.892	0.932**	1.21	0.901	0.974*	1.12	0.923
60 cm	0.907**	0.91	0.892	0.937**	0.46	0.914	0.954*	0.57	0.931	0.983**	0.31	0.947
100 cm	0.914**	0.61	0.923	0.647*	0.37	0.925	0.715*	5.15	0.641	0.746*	6.14	0.594
140 cm	0.621	9.16	0.461	0.513	9.26	0.598	0.681*	8.96	0.512	0.417	12.46	0.645

Based on our analysis of the soil water and heat relationship effects in the soil profiles, the coefficients of determination of the function models for the 40-, 60- and 100-cm layers for the BL were 0.851, 0.907 and 0.914, respectively. The correlation coefficient of the soil water and heat function models increased gradually within the 100-cm soil layer but decreased sharply to 0.621 in the 140-cm soil layer. Under the NS treatment, the coefficients of determination for the 20-, 40- and 60-cm soil layers were 4.43%, 6.59% and 12.35% greater, respectively, than that for the 10-cm soil layer. For the 100- and 140-cm soil layers, the coefficient of determination declined, and the fitting intensity effect between the two was weak. Similarly, as the soil depth increased under the CS and TS treatments, the coefficient of determination of the model also increased gradually as did the correlation between the soil moisture content and temperature. Similarly, the fitting effects of soil temperature and moisture in the 100- and 140-cm soil layers weakened under both conditions.

The effective coefficient (NSE) of the model was calculated based on the model’s prediction performance. With the increase in the coefficient of determination of the soil water and heat coupling model, the prediction performance improved and that the effective coefficient gradually increased. Under the BL condition, as the soil depth increased, the effective coefficient of the function models increased from 0.841 to 0.923 in the 20-, 40-, 60- and 100-cm soil layers. However, for the 140-cm soil layer, the function model’s accuracy was low; thus, the effective coefficient of the model decreased sharply to 0.461. Under the NS, the prediction error of the function model for the various soil layers was less than that under the BL. The snow cover enhanced the soil water and heat relationship, improving the model’s prediction accuracy. Similarly, under the CS and TS conditions, the model’s overall prediction error was lower than that observed under the BL. Furthermore, as the soil layers deepened, the prediction error of the model decreased, and the relationship effects of soil water and heat intensified. However, the model’s prediction error increased considerably in the 100- and 140-cm soil layers.

In frozen soil, soil moisture affected heat conduction, while temperature gradient promoted the migration of water, there was a strong relationship between soil moisture and temperature. Due to low thermal conductivity and large heat capacity of snow, snow cover hindered the effects of external environmental factors on the relationship between soil water and heat during the soil freezing period, the effect of isolating external environment was enhanced as the increase of snow cover thickness and density, and the relationship effects between soil moisture content and temperature increased. As the fitting intensity effects of the soil water and heat function models improved, its prediction performance were enhanced, and the model’s prediction error decreased. In vertical section, the energy transfer needs to pass through the soil medium layer. During this process, the uncertain environmental factors were gradually filtered out, and the effect of soil water and heat relationship in the frozen soil area was gradually enhanced. Combined with maximum freezing depths under the various treatment conditions, within the frozen layer, the soil water and heat relationship effects of the soil layers gradually increased as the soil depth increased. In contrast, outside the frozen layer, the soil water and heat interaction effects were weak, and the model’s prediction errors were larger. The overall performance was that the accuracy of the soil water and heat function models first increased and then decreased.

### Spatial variability of soil water and heat coupling during thawing

In the same way, we developed a soil water and heat function model by measuring the soil moisture content at point No. 1 and temperature in the 10-cm soil layer on multiple dates during the melting period. The soil temperature predicted by the function model at point No. 2 was compared with the temperature measured. The results showed in Table 5. It can be seen that the soil water and heat function models were less accurate when applied to the melting period than to the freezing period, but the variables of the function model passed a P < 0.05 significance test. For the BL treatment, the coefficient of determination (R^2^) of the function model was 0.871. However, for the snow cover treatments, the interaction relationship between the two decreased, and the coefficients of determination of models under the NS, CS and TS were 0.04, 0.258 and 0.34, respectively, less than that obtained under the BL. As the snow cover thickened, the interaction relationship between the soil moisture content and temperature gradually decreased.

**Table 5:** Relationship models of soil water and heat in the 10-cm frozen soil layer during the melting period.

	Treatment	Model of soil water and heat	Coefficient of determination, R^2^	P value	RE%	NSE
Melting stage	Bare land	*θ*_*V*_ = 13.8 exp(−((*T*_*s*_−9.64)/7.61)^2^)	0.871	0.031	3.65	0.859
	Natural snow	*θ*_*V*_ = −5.847 exp(3.57*T*_*s*_) + 11.76 exp(0.231*T*_*s*_)	0.831	0.049	4.13	0.836
	Compacted snow	*θ*_*V*_ = 8.019 exp(−4.17*T*_*s*_) + 9.017 exp(−1.624*T*_*s*_)	0.613	0.031	7.41	0.791
	Thick snow	${\theta }_{V}=0.19{T}_{S}^{4}+0.58{T}_{S}^{3}-0.94{T}_{S}^{2}-2.87{T}_{S}+16.87$	0.531	0.074	7.79	0.774

**Figure 5:**
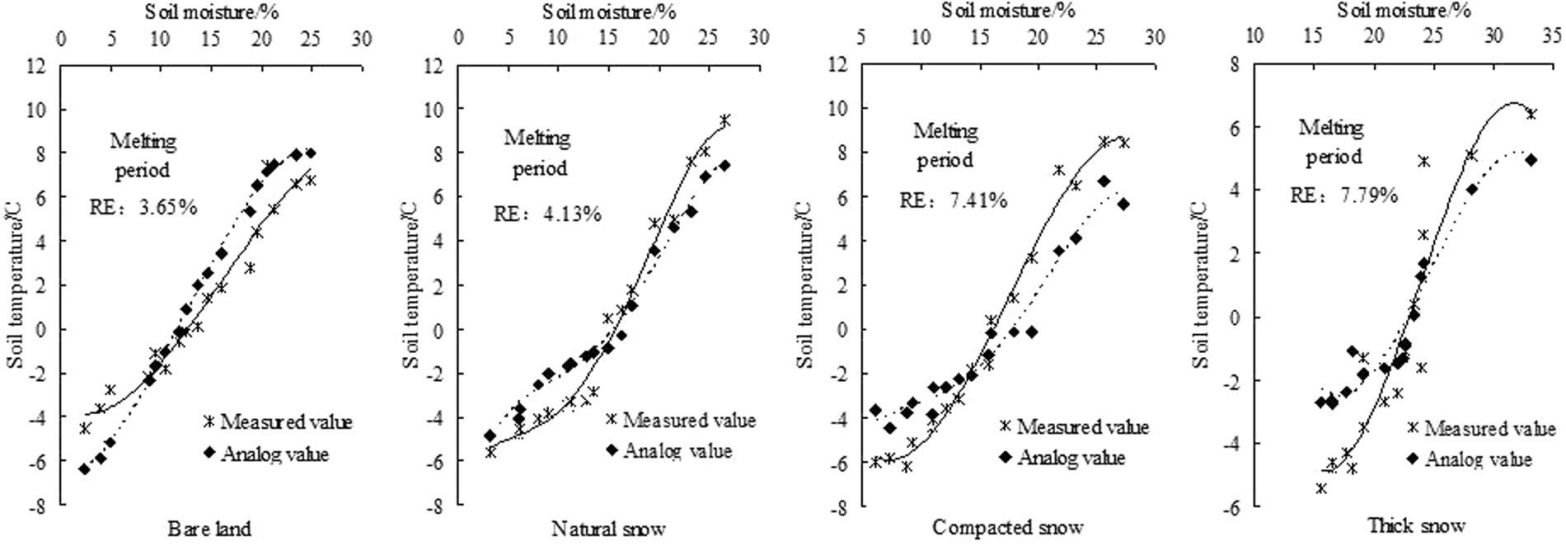
Comparison of values predicted by the soil water and heat coupling model and observed values in the 10-cm surface layer.

We analyzed the predictions of the soil water and heat function models under the four treatment conditions. It can be seen from the Fig. 5 that under the BL condition, the relationship effect between the soil water content and temperature was obvious, and as the ambient temperature increased, the soil moisture content and temperature increased steadily. After analyzing the predictions of the models, we noted that the NSE value was 0.859. Under the NS condition, when the soil temperature was less than 0 °C, the relationship effect between the soil temperature and water content was relatively strong. In contrast, when the temperature exceeded 0 °C, the interaction between the two was weaker, and the NSE of the model was lower. Furthermore, as the snow cover thickness and density increased, the NSE of the models for the CS and TS treatments decreased by 7.92% and 9.89%, respectively, relative to that of the BL treatment.

Based on the relationship effect of the 10 cm (surface) soil water and heat function model, we analyzed the relationship effect variations of the two in vertical profiles. The results are shown in Table 6. First, for the BL treatment, the coefficient of determination of the soil water and heat function model in the 20-cm soil layer was 0.904. The coefficients of determination of the model for the 40 cm and 60 cm soil layers were 2.21% and 4.64% greater than that for the 20-cm soil layer. The spatial variability in the soil water and heat relationship effects displayed patterns similar to those observed during the freezing period. In the 100-cm soil layer, the coefficient of determination of the soil water and heat function model decreased. Meanwhile, the relative error of the model increased from 0.49% to 3.82% within the 60–100-cm soil depth interval. During the thawing period, as the soil depth increased within the range of frozen activity, the accuracy of the model gradually improved. In contrast, the relationship effect outside of the frozen interval was poor.

**Table 6:** Effects of the soil water and heat relationship models under different treatments during the melting period. “**” denotes passing of the P = 0.01 significance test, and “*” denotes passing of the P = 0.05 significance test.

Soil depth	Bare land			Natural snow			Compacted snow			Thick snow		
	R^2^	RE/%	NSE	R^2^	RE/%	NSE	R^2^	RE/%	NSE	R^2^	RE/%	NSE
20 cm	0.904*	1.27	0.859	0.851*	2.54	0.843	0.773*	3.67	0.769	0.674*	4.87	0.763
40 cm	0.924**	0.79	0.877	0.882*	2.13	0.869	0.811*	2.37	0.826	0.791*	2.19	0.807
60 cm	0.946**	0.49	0.893	0.907**	0.59	0.871	0.841*	1.19	0.841	0.824*	1.14	0.832
100 cm	0.676*	3.82	0.642	0.531*	7.83	0.686	0.421*	10.97	0.596	0.551	6.25	0.564
140 cm	0.514	9.27	0.459	0.479	10.76	0.571	0.317	8.64	0.421	0.403	9.79	0.467

Under the NS treatment, as the soil depth increased, the coefficients of determination of the function models in the 20-, 40-, and 60-cm soil layers were consistent with those observed under the BL and exhibited an increasing trend. The coefficients of determination of the models in the 20-, 40- and 60-cm soil intervals under NS were 5.86%, 4.54% and 4.12% lower, respectively, than those in the same intervals under the BL. The relationship effects yielded by the models were less under the snow cover, and the models’ relative errors for these plots were greater than those under the BL.

Similarly, analysis of the relationship effects spatial variations under the CS and TS treatments showed that it first increased from 20 cm to 140 cm and then abruptly decreased when passing into the frozen layer. Based on the coefficients of determination of the function models for a given soil interval under the various snow cover conditions, as the snow cover thickness and density increased, the accuracy of the function models gradually decreased. In addition, the NSE of the soil model decreased, and the models’ prediction performance declined. Based on general energy transfer patterns in snow-covered farmland soil, due to infiltration of snowmelt, the large specific heat capacity of water inhibits temperature changes, thereby affecting the relationship of the soil temperature and moisture content, this effects became more pronounced as the snow cover thickened.

In summary, during melting period, solid snow was converted to liquid water, a large number of water infiltration affected the temperature gradient changes, at the same time, the change of temperature gradient reacted to the migration of water, snowmelt affected interactions relationship between soil temperature and moisture content to a certain extent, thus, the relationship effect between the soil temperature and moisture content declined with the increase of snow cover. In addition, the accuracy of the soil water and heat function models gradually increased with increasing depth within the soil freeze activity zone. However, the soil water and heat function effects were weaker than those during freezing periods. Our analysis of the soil during the melting period revealed that two-way soil melting also affected the soil water-heat interactions relationship.

## Discussion

1. The results of the manuscript are derived from field monitoring data. First, the variations in soil moisture content and temperature in different soil layers on vertical profile were analyzed. On this basis, the soil water and heat coupling equations were developed, and the errors between the measured values and those predicted by the model were analyzed to verify the coupling effects of the models. Variations in the results produced by the soil water and heat coupling models across the soil profile and in relation to the depth of the frozen soil were explained. We observed the patterns of soil water and heat transfer under the various snow cover conditions and the coupling relationship between the two parameters. However, the underlying physical mechanisms and quantification of the transfer relationships within the atmosphere-snow-soil system require further study.
2. In the course of the experiment, with the reduction of ambient temperature, the soil moisture content and temperature were affected by environmental factors, and the changes in soil moisture content and temperature showed a pattern of initial decrease followed by stability and then increase. Although the variations in soil moisture and temperature differed substantially between the soil intervals, with increasing depth, the energy transfer in the soil accompanied by the phenomenon of loss and the force of soil hydrothermal driven by the external environment weakened. It was proved by experiment that under the BL condition, the range (ΔT) of soil temperatures was 18.9 °C at the surface (10 cm) soil layer, and the change in soil water content was 19.51%. However, with increasing depth, the change was small. In the 140-cm layer, the range of temperature was 8.9 °C, whereas the range of water content was only 3.66%. As Jame *et al*.^13^ reported that as the soil moisture migrated downward with the frozen front and heat transfer occurred during freezing, the moisture content and temperature of the frozen soil layers decreased. Fan *et al*.^33^ studied the water and temperature characteristics of a soil profile in an alpine grassland area of northern Tibet. They reported that the soil moisture and temperature displayed periodic fluctuations with variations in the ambient temperature, and there were clear hysteresis patterns and decreased variations with increasing depth. In addition, snow cover hindered energy exchange between the soil and the environment, and overall fluctuations of soil water and heat under three types of snow cover were weakened. Maurer *et al*.^34^ concluded that soil temperature and moisture were strongly affected by seasonal snow cover, and soil water content and heat under snow cover were greater than under bare soil.
3. In the soil water-heat system, the changes in soil temperature affected the phase changes of soil water. The migration of soil moisture accompanied by heat conduction affected the soil temperature variations, and there was a strong coupled interaction between the two parameters. Due to the special physical properties of snow deposits, it restrained the influence of the external environment on the soil water heat and stabilized the relationship between the two parameters. The soil water and heat function model performed well under the BL, and the corresponding prediction error was 6.19%. Under the snow cover, the relationship effects between soil moisture and temperature increased and the prediction accuracy of the model improved with increasing snow cover thickness and density. As Chang^35^ reported, land cover was conducive to maintaining the stability of soil moisture and temperature and maintaining a strong interaction between the two variables. During the melting period, the infiltration of snowmelt supplemented the surface moisture content and affected the regular change in soil temperature. The data showed that the prediction error of the model under the snow cover treatment was greater than that of the BL treatment, and with increasing snow thickness, the relationship effect gradually weakened. As Wang^36^ reported, the soil was affected more by the environment and snow melting during the melting stage, the soil moisture and temperature varied more randomly, and the interaction between the two variables was weaker.
4. In the vertical section of soil, soil moisture and temperature were affected by the environment, but soil water and heat migration stabilized with increasing soil depth, i.e., the changes at deep layer were less than those in the surface soil interval. Under the snow cover treatments, the accuracy results of the soil water and heat function models improved with increasing depth within the zone of soil freezing. However, the performance of the model effects were weaker outside the frozen zone, and the prediction error of the models were large. The results of the function models showed a trend of initial increase and subsequent decrease in the vertical profile. As Wang^37^ pointed out, in the process of soil freezing - thawing circulation, the heat flux of deep soil was relatively low, and the freezing rate of soil slowed down with the delay of freezing time and the expansion of freezing depth. The influence of meteorological factors fluctuations on the soil was weak, and the correlation between soil moisture and temperature gradually increased.

## Conclusions

1. During soil freezing and thawing periods, as the ambient temperature decreased, frozen fronts in the soil migrate downward, and the soil temperature and water content first decreased and then increased. As the soil depth increased, ranges of soil moisture and temperature change gradually decreased and were less disturbed by sudden changes in the environment. During freezing period, the large thermal capacity and high albedo of snow ensured the conservation of soil energy. In the 10-cm layer, temperature fluctuations under the NS, CS and TS treatments were 5.07, 7.73 and 9.51 °C less, respectively, than those under the BL treatment. Apart from this, the variability of soil moisture content under freezing condition was relatively low. During the melting period, snowmelt water had a certain infiltration effect, which leaded to a large increase in soil moisture content, and affected the steady change trend of soil temperature and water content
2. In the process of soil freezing thawing periods, there was a certain response relationship between soil moisture content and temperature, and this relationship accorded with exponential filtering model. During freezing period, snow cover was a porous medium that hindered environmental effects on soil water and temperature, and the models of soil water and heat relationship under snow cover were accurate. In the 10-cm (surface) layer, the model’s relative error (RE) for the NS treatment was 0.52% less than that of the BL treatment. Additionally, as the snow cover and density increased, the model’s RE decreased gradually. During melting period, snowmelt infiltration affected the stability of the soil water content, the relationship between soil temperature and moisture was gradually weakened.
3. Regarding the spatial variability of soil water and heat regression function model effect, within the soil freezing range, as the soil depth increased, the model fitting accuracy and prediction effects of the soil water and heat function models gradually increased, it means that the relationship between soil temperature and moisture was gradually increased, and beyond the freezing zone, the coefficients of determination of the coupling models were greatly reduced. Furthermore, the relative errors of the models first decreased and then increased with increasing depth. The relationship effects reflected by the soil water and heat function models under snow cover conditions were greater than those under non-mulching treatments during freezing period, and this advantage became more obvious as the snow cover thickened. Meltwater infiltration and two-way melting of soil affected soil water and heat interactions during the snowmelt period, and the soil water and heat relationship effects were gradually weakened under snow cover.
4. Based on the above research, the fitting function model constructed in this paper characterized the trend of gradual decrease of water content with decreasing soil temperature in seasonally frozen soil, it reflected the relationship between moisture and temperature in freeze-thaw soil. In the fitting process of the function, the fitting accuracy of the function model accorded with the specification requirements, and its prediction error was within the allowable range. Therefore, the function model is suitable for quantitative transfer characterization of moisture and temperature of black loam in the process of freezing and thawing. However, the disturbance of the external environment will affect the fitting accuracy and prediction effect of the function model to a certain extent. Therefore, it is necessary to continuously optimize its model parameters in future studies to ensure that it adapts to different surface coverage conditions, thereby enhancing the accuracy of the transfer function.
